# Population divergence in compensatory growth responses and their costs in sticklebacks

**DOI:** 10.1002/ece3.1342

**Published:** 2014-12-03

**Authors:** Nurul Izza Ab Ghani, Juha Merilä

**Affiliations:** 1 Ecological Genetics Research Unit, Department of Biosciences, University of HelsinkiPO Box 65, FI-00014, Helsinki, Finland; 2 Faculty of Science, Department of Biology, University of Putra Malaysia43400 UPM Serdang, Selangor Darul Ehsan, Malaysia

**Keywords:** Body size, compensatory growth, population differentiation, stickleback

## Abstract

Compensatory growth (CG) may be an adaptive mechanism that helps to restore an organisms’ growth trajectory and adult size from deviations caused by early life resource limitation. Yet, few studies have investigated the genetic basis of CG potential and existence of genetically based population differentiation in CG potential. We studied population differentiation, genetic basis, and costs of CG potential in nine-spined sticklebacks (*Pungitius pungitius*) differing in their normal growth patterns. As selection favors large body size in pond and small body size in marine populations, we expected CG to occur in the pond but not in the marine population. By manipulating feeding conditions (*viz*. high, low and recovery feeding treatments), we found clear evidence for CG in the pond but not in the marine population, as well as evidence for catch-up growth (i.e., size compensation without growth acceleration) in both populations. In the marine population, overcompensation occurred individuals from the recovery treatment grew eventually larger than those from the high feeding treatment. In both populations, the recovery feeding treatment reduced maturation probability. The recovery feeding treatment also reduced survival probability in the marine but not in the pond population. Analysis of interpopulation hybrids further suggested that both genetic and maternal effects contributed to the population differences in CG. Hence, apart from demonstrating intrinsic costs for recovery growth, both genetic and maternal effects were identified to be important modulators of CG responses. The results provide an evidence for adaptive differentiation in recovery growth potential.

## Introduction

Spatial and temporal variations in resource levels are of commonplace occurrence in nature, and individuals born in times when resource levels are low can face considerable challenges during early growth and development. Lowered food intake is known to reduce early growth and development in variety of organisms (Calder 1984; Sebens 1987; Dmitriew 2011) and can translate to delayed maturation at a smaller size (Metcalfe and Monaghan 2001). Delayed maturation in turn may decrease fitness by increasing generation time and decreasing reproductive life span (Roff 1992; Stearns 1992). Likewise, small adult size may directly reduce fitness because both survival probability and reproductive success tend to increase with increasing body size (Roff 1992; Stearns 1992). Consequently, it is reasonable to expect that natural selection should favor the evolution of mechanisms mitigating negative fitness consequences of early life growth limitation, whether resulting from food restriction or some other unfavorable environmental condition.

Compensatory growth (CG) is a form of growth plasticity in which growth accelerates to catch-up to the original growth trajectory once favorable conditions are restored after a period of growth depression (Metcalfe and Monaghan 2001; Ali et al. 2003). If heritable, the potential for CG responses is expected to evolve in populations that are subject to predictable variations in resource availability, in populations where fitness loss due to growth depression is strongly selected against and/or in populations where the costs of compensatory growth responses can be delayed (Yearsley et al. 2004; Mangel and Munch 2005; Fraser et al. 2007; Lee et al. 2011). However, these predictions have seldom been evaluated empirically, and as far as we are aware, only four studies have compared CG responses among different populations of the same species (Purchase and Brown 2001; Schultz et al. 2002; Álvarez and Metcalfe 2007; Fraser et al. 2007). Interpretations of these results, as well as those of CG studies in general, are further complicated by semantic confusion surrounding the definition of CG (Jobling 2010), as well as methodological problems that plague studies of CG responses (Nicieza and Álvarez 2009; Jobling 2010). In particular, few of the many studies focussed on CG (reviewed in Ali et al. 2003) have controlled for negative size dependency in growth responses. Likewise, the growth acceleration following a period of growth depression and subsequent restoration of favorable conditions may not be anything more than resumption of normal growth of initially starved and hence small-sized individuals (i.e., catch-up growth, Nicieza and Álvarez 2009). Furthermore, and not surprisingly given the above-mentioned problems, little is known about the relative importance of additive genetic, nonadditive genetic, and maternal effect influences on variation in CG responses.

Marine and pond populations of the nine-spined stickleback (*Pungitius pungitius*) provide an interesting model system for studies of CG responses. The marine ecotype matures at an early age and small size, whereas the pond ecotype exhibits delayed maturation at large size (Herczeg et al. 2009; Shimada et al. 2011; Ab Ghani et al. 2012, 2013a). There is an direct (e.g., Shimada et al. 2011; Karhunen et al. 2014) and indirect (reviewed in Merilä 2013) evidence to suggest that reaching a large size is under strong positive selection in ponds lacking piscine predators, whereas the opposite is likely to be true in the sea. Comparisons of pond and marine populations show that marine fish grow faster than the pond fish (Herczeg et al. 2012; Ab Ghani et al. 2013b; Aikio et al. 2013), and that pond fish continue their growth longer to reach a larger final size at maturation (Shimada et al. 2011; Herczeg et al. 2012). However, whether these two-nine-spined stickleback ecotypes have diverged in their ability to mount CG responses once released from food restriction is currently unknown.

Based on the observation that stronger CG growth responses are associated with high routine growth rates in other species (Schultz et al. 2002; Fraser et al. 2007), one might expect to find stronger CG responses among marine than among pond nine-spined sticklebacks. However, given that compensatory responses are likely to require increased activity and movements which increase the risk of being eaten up by predators (e.g., Gotthard 2000; Biro and Stamps 2008), one might also expect the opposite as marine fish cohabitate with various predators. Therefore, high predation risk provides a good reason to expect reduced CG response to food deprivation in the marine populations. In fact, experimental evidence shows that marine *P. pungitius* reduce their growth in response to predation more than the pond fish (Välimäki and Herczeg 2012). Moreover, as fitness loss due to stunted growth is likely to be higher for pond than for marine fish (Herczeg et al. 2010), CG responses can be expected to be stronger for pond than for marine fish. An additional reason to expect stronger CG responses in the pond ecotypes relates to the ecology of ponds: the high-latitude ponds are strongly seasonal habitats where the yearly window of opportunity for growth is more limited and unpredictable than that in more stable marine habitats. In addition, periods of food shortage may occur in ponds because the population densities – and thereby also the degree of intraspecific competition for food – may fluctuate widely and therefore select for the ability to mount CG responses when feeding conditions improve (*cf*. Mangel and Munch 2005). Overall, there is more reason to expect stronger CG responses in pond as compared to marine nine-spined sticklebacks.

The aim of this study was to investigate the existence and magnitude of CG responses in an interpopulation context and explore the possible costs of such responses in terms of individuals’ intrinsic survival and maturation probabilities. Apart from testing for population differences in CG responses and their costs, we also looked for evidence of the nature (*cf*. additive, nonadditive) of genetic variation in these responses. To this end, we conducted a common garden experiment using *P. pungitius* from two populations known to differ in their growth rates (Herczeg et al. 2012; Ab Ghani et al. 2013b) and sizes at maturation (Ab Ghani et al. 2012). To manipulate growth, individually grown fish were exposed to high, low, and recovery feeding treatments, the latter of which consisted of a period of low feeding followed by ad libitum feeding. To study the genetic basis of recovery responses, reciprocal interpopulation “hybrid” crosses alongside “pure” marine and pond population crosses were utilized. We hypothesized that if variation in CG responses between populations is due to additive genetic effects, the “pure” crosses will differ in their responses, while the “hybrids” will be intermediate to the “pure” crosses in their responses (*cf*. Ab Ghani et al. 2012, 2013a). In the case that nonadditive genetic or maternal effects are of influence, the “hybrids” are expected to deviate from the intermediacy between the pure crosses. In the case of simple dominance, individuals from both “hybrid” crosses are expected to deviate toward the mean of the “pure” cross that is carrying the dominant allele(s). Likewise, if maternal effects are of influence, individuals from both “hybrids” are likely to deviate from the intermediacy toward their mothers’ “pure” cross means. Finally, we hypothesized that if the CG responses are costly, we should see an increased incidence of mortality and delayed timing of maturation among individuals exposed to recovery as compared to the high feeding treatment.

## Materials and Methods

### Study populations and materials

Adult *P. pungitius* were collected during early breeding season (late May to mid-June) of 2010 from a Baltic Sea (Helsinki: 60°12′09″N, 25°10′58″E) and a pond (Pyöreälampi: 66°15′40″N, 29°26′00″E) site to be used as broodstock for F_1_ common garden fish, which were produced through artificial fertilizations. Fish from these geographically distinct (∼900 km apart) sites are phenotypically (Herczeg et al. 2009, 2010; Shimada et al. 2011; Ab Ghani et al. 2012) and genetically (*F*_ST_ = 0.46: Shikano et al. 2010; *Q*_ST_ > 0.90 for body size, Shimada et al. 2011) divergent. The small-sized marine fish (total length <5 cm) were caught using a seine net from a shallow coastal, brackish water bay (salinity 0–6.0 psu, Shimada et al. 2011) representing a heterogeneous habitat where *P. pungitiu*s is sympatric with a large number of predatory and competitor fish. The pond fish (total length occasionally >11 cm) were caught using minnow traps from a freshwater pond (surface area of <5 ha) representing a homogeneous habitat where *P. pungitiu*s is the only fish species apart from introduced whitefish (*Coregonus lavaretus*).

The artificial fertilizations were made in vitro between randomly chosen males and females at the site of capture (Pyöreälampi fish) or in the laboratory (Helsinki fish and the hybrids). Although the conditions for the fertilized eggs were not fully identical for all cross-types during the first two days of their development, earlier analyses have confirmed that this did not influence the subsequent development of eggs and larvae (Ab Ghani et al. 2012). The artificial fertilizations were made by pouring sperm solution – obtained by mincing the testicles of overanaesthetized males in a drop of water – over eggs. The eggs were obtained by gently squeezing the ripe females. Four different cross-types were produced: two “pure” crosses by crossing either Helsinki males with Helsinki females (hereafter the marine, MM) or Pyöreälampi males with Pyöreälampi females (hereafter the pond, PP), and two “hybrid” crosses using either Helsinki males with Pyöreälampi females (hereafter MP) or Pyöreälampi males with Helsinki females (hereafter PM). In total, 40 full-sib families (ten per cross-type) were produced, and each parent was used for only one cross.

### Growth conditions and feeding treatments

A total of 400 fish (ten individuals/family) were reared individually in 1.4-L tanks housed in four Zebrafish Rack Systems (Aquaneering Inc., San Diego, CA, USA) equipped with physical, biological, and UV filters. Visual contacts between individuals were blocked by panels placed between tanks. Individual rearing ensured that social interactions were not confounding the observed effects and interpretations (*cf*. Zhu et al. 2004). All fish were kept in 0 psu salinity under a 14 : 10 h light:dark photoperiod and 17°C water temperature from 1 until 299 days after hatching (hereafter DAH). At 300 DAH, all fish were subjected to artificial wintering (to facilitate reproduction for other scientific purposes), during which the photoperiod was gradually shifted toward 24-h dark and the temperature toward 4°C over a two-week period. The wintering lasted for 30 days, after which water temperature and photoperiod were gradually increased back to 17°C and 24-h light. All fish were kept under these conditions for 97 days (i.e., until 441 DAH) before they were subjected to a second artificial wintering, following the protocol described above. The experiments were terminated at 510 DAH.

Seven days were required for fish to hatch from eggs. Thus, fish were reared in one of the three different feeding treatments: high, low, and recovery treatments which started at 7 DAH. In the high feeding treatment, fish were fed *ad libitum* two times per day, whereas in the low feeding treatment, they were fed *ad libitum* once every two days. In the recovery feeding treatment, fish were subjected first to the low feeding treatment between 7 and 90 DAH and then switched to the high feeding treatment at 91 DAH. A total of 200 fish were reared in high feeding treatment (50 individuals/cross-type), 100 fish in the low feeding treatment (25 individuals/cross-type), and 100 fish in the recovery feeding treatment (25 individuals/cross-type). All fish were fed with live brine shrimp (*Artemia* sp.) nauplii for the first two months and with frozen bloodworms thereafter. A two-week adjustment period was employed before switching food from *Artemia* sp. to bloodworms, during which the fish were fed with a 1:1 mixture of *Artemia* sp. and chopped frozen bloodworms. After this, fish were fed with chopped frozen bloodworms for another 2 weeks. At 75 DAH onwards, fish were fed with whole frozen bloodworms.

### Size and growth measurements

Standard length (SL), measured from the tip of the lower jaw to the base of the caudal peduncle, was recorded to the closest 0.01 mm from photographs taken of each fish at 15 different time points (30, 60, 90, 120, 150, 180, 210, 240, 270, 330, 360, 390, 420, 480, and 510 DAH) using the program TPSDIG 2 (Rohlf 2002). All individuals were photographed (alive) using a digital camera (Nikon D60), with a ruler placed as a size reference in each photograph. The data set of size at 510 DAH was comprised of 126 individuals from high feeding treatment (MM: 25, MP: 33, PM: 24 and PP: 44), 35 individuals from low feeding treatment (MM: 12, MP: 12, PM: 6 and PP: 5), and 66 individuals from recovery feeding treatment (MM: 4, MP: 14, PM: 24 and PP: 24).

To allow comparison of growth rates among treatments, we calculated specific growth rates (*SGR*) using the equation (e.g., Nicieza and Álvarez 2009): 

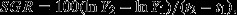
1where ln *Y*_1_ refers to ln transformed (initial) size at time point *t*_1_, and ln *Y*_2_ refers to ln transformed size at time point *t*_2_. As *SGR* (also known as instantaneous relative growth rate) shows negative size dependency (e.g., Jobling 2010),and the mean size of individuals in different treatments differed at the time the recovery feeding was initiated (see Results), direct comparisons among treatments could be confounded by initial size differences (Nicieza and Álvarez 2009). Therefore, we used linear models (see below) to control for size dependency in *SGR* by including initial size (at the beginning of given growth period) as a covariate into the models. Although this “synchronous” approach should provide a fairly robust way to make growth rates size independent, there is a risk of spurious correlation as the covariate (initial size; *Y*_1_) is involved also with the response variable (*SGR*; Nicieza and Álvarez 2009). Thus, we also analyzed absolute growth increments (*k*) obtained as the simple (logarithmic) difference between body size measurements at two points: 

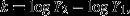
2where log *Y*_1_ denotes as log-transformed of size at time *t*_1,_ and log *Y*_2_ denotes as log-transformed size at time *t*_2_. This measure is also size dependent so comparisons between treatments require accounting for differences in initial size at the beginning of the given growth period (i.e., time point *t*_1_). This was accomplished by adding (Log) *Y*_1_ as a covariate in the analyses conducted using linear models. Note that this ANCOVA of absolute growth increments is equivalent to repeated measures of ANOVA of body size over a single time interval, and the treatment × repeated measures interaction provides a reliable test for compensatory growth (Nicieza and Álvarez 2009). Application of “asynchronous approach” (*cf*. Nicieza and Álvarez 2009) verified occurrence of compensatory growth in our data (Appendix 1).

### Survival analyses

Survival was monitored and recorded throughout the experimental period, but the analyses were divided to two time intervals: (1) before (91–510 DAH); and (2) after initiation of recovery feeding treatment (91– 510 DAH). In the first case, all individuals that died before 90 DAH were recorded as zeros, while the survivors were recorded as 1's. Likewise, in the second analysis, all individuals that were alive at 91 DAH but died before 510 DAH were recorded as zeros, while all individuals surviving until 510 DAH were recorded as 1's. Precise mortality date for all deaths was also recorded. The initial sample size was 400 fish for (1) and 352 for (2). Detailed information about sample sizes in different treatments in different phases of the experiments is available from Appendix 2.

### Timing of maturation

Timing of maturation was recorded starting from the day when the first artificial wintering ended (344 DAH) and continued until 510 DAH. During this time interval, records were available for 288 individuals alive on 344 DAH (see Appendix 2 for detailed sample sizes) which included both mature and immature individuals. The date of reaching maturation was recorded based on phenotypic criteria (see below), and all of the mature individuals were scored as 1's, while all immature individuals were scored as zeros. Maturation was judged on the basis of visual inspection of male secondary sexual characters or the presence of eggs in females as explained in Ab Ghani et al. (2013a). Immature individuals lacking diagnostic phenotypic criteria were sexed using molecular methods following Shikano et al. (2011) and Ab Ghani et al. (2013a).

### Statistical analyses

A general linear mixed model (GLMM) was used to evaluate the body size differences among different cross-types, sexes, and feeding treatments (all fixed factors) at the time of the last observation (510 DAH), using PROC MIXED (Littell et al. 2006) with family nested within cross-type. The significance of the pairwise comparisons among cross-types and treatments was evaluated after false discovery rate (FDR) adjustment (Benjamini and Hochberg 1995).

Growth curves were plotted for illustrative purposes using actual mean sizes (SL) at given ages for all cross-types in different feeding treatments (Fig. 2). To analyse the relative influence of feeding treatment, cross-type, and sex on mean body size, a repeated measures GLMM was implemented with body size as a dependent variable, feeding treatment, cross-type, and sex as fixed factors. Repeated measures of mean body size at different measurement time points were treated as a repeated measures factor and family nested within cross-type as a random factor. All two- and three-way interactions between fixed factors and the single explanatory variable were included in the initial model. Akaike Information Criterion (AIC) identified compound symmetric covariance structure as the best fitting for the data (*cf*. Littell et al. 2000).

The relative influence of feeding treatment, cross-type, and sex on *k* between 91 and 120 DAH was analyzed with a GLMM fitting feeding treatment, cross-type, and sex as fixed factors, and size at 90 DAH as a covariate. Family was nested within cross-type as a random factor. This analysis was complemented by a repeated measures GLMM where sizes at 90 and 120 DAH were treated as response variables, treatment, cross-type and sex as fixed factors, and family as a random effect (nested with cross-type). All GLMM analyses were performed using the SAS 9.2 (SAS Institute Inc 2007) software package, and a backward stepwise model selection based on the *P *<* *0.05 criterion was applied as it is considered to be a conservative method (Murtaugh 2009). We started with the full factorial models and then removed the nonsignificant terms, starting with the highest level interactions and ending with the main effects. The main effects (and lower order interactions) included in significant interactions were not removed.

The survival and maturation probability analyses were carried out using the Survival Kit v.6 (Ducrocq et al. 2010) which is capable of handling mixed-model analyses with random effects and censored data. The probability of survival was modeled in Cox regression, and separate models were fitted for data before and after implementation of the recovery feeding treatment. Feeding treatment, cross-type, sex,and their interactions were used as predictors, and family within cross-type was treated as a random factor. *SGR* between 31 and 60 DAH was used as a covariate to evaluate the influence of growth rate on the probability of survival before the initiation of the recovery feeding treatment. The probabilities of survival and maturation after the recovery feeding treatment utilized model otherwise similar to the model described above, but the *SGR* between 91 and 120 DAH was added as a covariate to evaluate the influence of growth rate on the probability of survival and/or maturation. For a finer resolution, we also ran pairwise Cox regressions between different cross-types and feeding treatments, separately for both before and after recovery feeding treatment. The significance of the results was evaluated after FDR adjustment (Benjamini and Hochberg 1995).

The primary data underlying this publication have deposited to Dryad (doi:10.5061/dryad.40r32).

## Results

### Final body size

**Figure 1:**
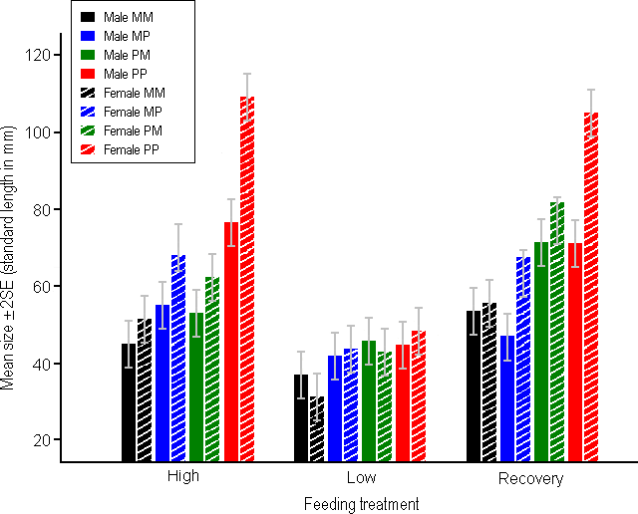
Mean body size (± SE) of male and female nine-spined sticklebacks in different feeding treatments and crosses at the end (510 DAH) of the experiments. M = marine, P = pond. For the hybrids, the first abbreviation denotes origin of father, the second origin of mother.

Mean size of the fish at the end of the experiment (510 DAH) was significantly influenced by feeding treatment (*F*_2,379_ = 80.28, *P* < 0.001), cross-type (*F*_3,54_ = 28.54, *P* < 0.001), and sex (*F*_1,383_ = 7.94, *P* < 0.01). In general, individuals from the low feeding treatment reached a smaller size than those from the high or recovery feeding treatments (Fig.1). Furthermore, females were generally larger than males (Fig.1) albeit the degree of sex difference was less in the low as compared to the high and recovery feeding treatments (sex × feeding treatment interaction: *F*_2,381_ = 8.76, *P* < 0.001; Fig.1). Likewise, although the cross-type-specific differences in size were largely similar across the different feeding treatments, the magnitude of these differences was less pronounced in the low as compared to high and recovery feeding treatments as indicated by the significant feeding treatment × cross-type interaction (*F*_6,379_ = 10.55, *P* < 0.001). All other interactions were nonsignificant (*F* ≤ 0.57, *P* > 0.63), as was the random effect of family (*z* = 0.61, *P* = 0.27).

### Patterns of growth

**Figure 2:**
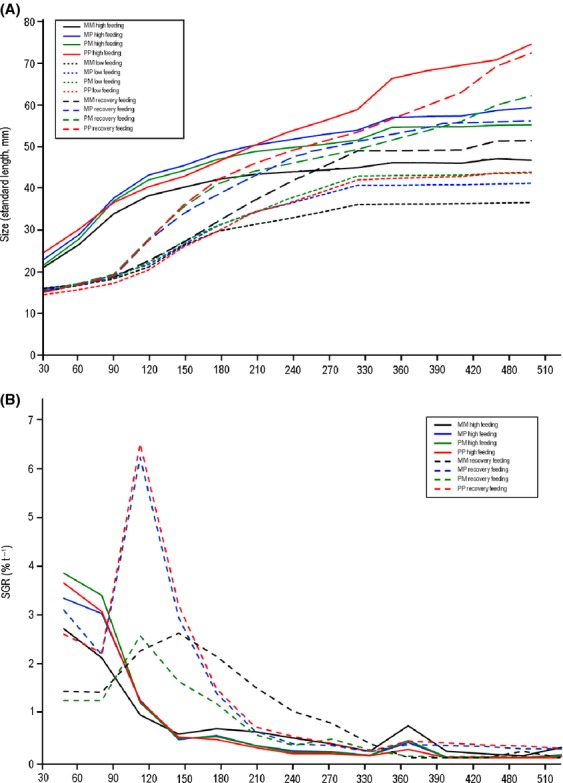
Growth trajectories and growth rates of four cross-types of nine-spined sticklebacks in different feeding treatments as function of time. (A) Mean growth trajectories fitted through actual mean body size measurement in high, low and recovery feeding treatments separately for each cross-type. (B) Specific growth rates (*SGR*) at different time intervals calculated from the data shown in (A). M = marine, P = pond. For the hybrids, the first abbreviation denotes origin of father, the second origin of mother.

The general patterns of growth leading to the above-described differences in final size differed greatly between different feeding treatments (Fig. 2A). Throughout the experiment, fish from the high feeding treatment were larger than fish from the low feeding treatment in all four cross-types (Fig.2A). The fish subject to the recovery treatment showed a distinctively different pattern: when the recovery feeding was initiated (91 DAH), fish from all but the pure marine crosses (MM) experienced a fast size increase and caught up eventually with the fish from the high feeding treatment (Fig.2A). Notably, also the marine fish (MM) that showed a slow initial growth response in the recovery feeding treatment caught up eventually with the MM fish from the high feeding treatment, and at the end of the experiment, all the crosses from the recovery treatment showed either full or over compensation (Figs.1, 2A). Pairwise comparisons of mean sizes of the different cross-types between treatments gave quantitative support for these observations (Appendix 3). Namely, within each cross-type, mean size of fish in the low feeding treatment was significantly smaller than that of the fish from high or recovery feeding treatments, whereas in the case of two cross-types (MM, PM), mean size of the fish from the recovery treatment significantly exceeded that of the fish in the high feeding treatment (i.e., over compensation; Fig. 1; Appendix 3). In the two other cases (PP, MP), there was no difference among recovery and high feeding treatment fish (i.e., full compensation; Fig. 1; Appendix 3).

A repeated measures GLMM gave quantitative support for the qualitative descriptions above: all the main effects – including the repeated measures factor – were significant (Table1), and portrayed the differences detected in the analysis of final size (510 DAH) above. However, all of the two-way and most of the three-way interactions were also significant (Table1), which is not surprising given the heterogeneity in growth trajectories as depicted in Figure2A. However, it is noteworthy that all interactions involving the (time dependent) repeated measure factor were highly significant (Table1), supporting the impression emerging from Figure2A that patterns of growth differed among feeding treatments, cross-types, and even between the sexes (Fig.1).

**Table 1:** Results of the repeated measures general linear mixed model of body size (SL at different time points) of male and female nine-spined sticklebacks from four cross-types in three feeding treatments (high, low & recovery). Family was included as a random factor. df_1_ = numerator degrees of freedom, df_2_ = denominator degrees of freedom

Source	df_1_, df_2_	*F*	*P*
Feeding treatment	24,311	2819.22	< 0.0001
Cross-type	34,313	50.92	< 0.0001
Sex	14,313	58.86	< 0.0001
Repeat (time)	144,282	1848.75	< 0.0001
Feeding treatment × cross-type	64,312	55.13	< 0.0001
Feeding treatment × sex	24,310	88.81	< 0.0001
Feeding treatment × repeat	284,282	44.59	< 0.0001
Cross-type × sex	34,313	5.74	0.0006
Cross-type × repeat	424,282	20.50	< 0.0001
Sex × repeat	144,281	2.03	0.0125
Feeding treatment × cross-type × sex	64,308	11.96	< 0.0001
Feeding treatment × cross-type × repeat	844,282	4.23	< 0.0001
Feeding treatment × sex × repeat	284,281	4.66	< 0.0001

### Testing for compensatory growth

Specific growth rates (*SGR*s) in the high feeding treatment declined in time for all cross-types: rapid initial growth levelled off by 150 DAH and remained low thereafter (Fig.2B). However, *SGR*s in the recovery feeding treatment showed a distinctive increase following the administration of the recovery feeding (90 DAH) and remained higher than *SGR*s for high feeding treatment until 180–330 DAH (Fig.2B). In particular, *SGR*s for fish having pond mothers (PP & MP) displayed a strongly elevated *SGR* following the administration of the recovery feeding, whereas those with marine mothers (MM & PM) responded less strongly (Fig.2B).

A GLMM focussed on *SGR* over the 91–120 DAH growth interval (the period of rapid growth following release from food restriction; Fig. 2A) and controlling for initial size differences among subjects verified that compensatory growth occurred (Table2). Namely, apart from significant effects of feeding treatment and cross-type on *SGR*, the feeding treatment × cross-type interaction was also significant, showing that compensatory growth response was present in some (PP & MP; Fig.2B), but not in all crosses (Table2). Furthermore, the three-way interaction between feeding treatment, cross-type, and sex indicated that the response was stronger in females than among males in the crosses in which it occurred (Table2). The conclusions remained unchanged if the analyses were conducted using absolute growth increments (*k*) controlling for initial size (GLMM; feeding treatment: *F*_1,235_ = 4.38, *P* = 0.0135; feeding treatment × cross-type interaction: *F*_3,212_ = 3.81, *P* = 0.0109; Appendix 4), or if a repeated measures GLMM was utilized (feeding treatment × repeated measures: *F*_3,494_ =11.29, *P* < 0.0001; Appendix 5).

**Table 2:** GLMM of *SGR* 91–120 DAH among four cross-types of *Pungitius pungitius* in high and recovery feeding treatments SL90 = standard length at 90 DAH. df_1_ = numerator degrees of freedom, df_2_ = denominator degrees of freedom

Source	df_1_, df_2_	*F*	*P*
Feeding treatment	1,238	16.48	< 0.0001
Cross-type	1,238	16.48	0.0009
Sex	1,239	2.35	0.1267
SL90	1,240	17.90	< 0.0001
Feeding treatment × cross-type	3,204	5.40	0.0014
Feeding treatment × sex	1,237	2.30	0.0848
Feeding treatment × SL90	1,235	7.65	0.0061
Cross-type × sex	3,238	4.00	0.0084
Cross-type × SL90	3,203	4.401	0.0050
Sex × SL90	1,239	2.66	0.1041
Feeding treatment × cross-type × sex	3,235	4.04	0.0080
Cross-type × sex × SL90	3,237	3.89	0.0097

### Survival

**Figure 3:**
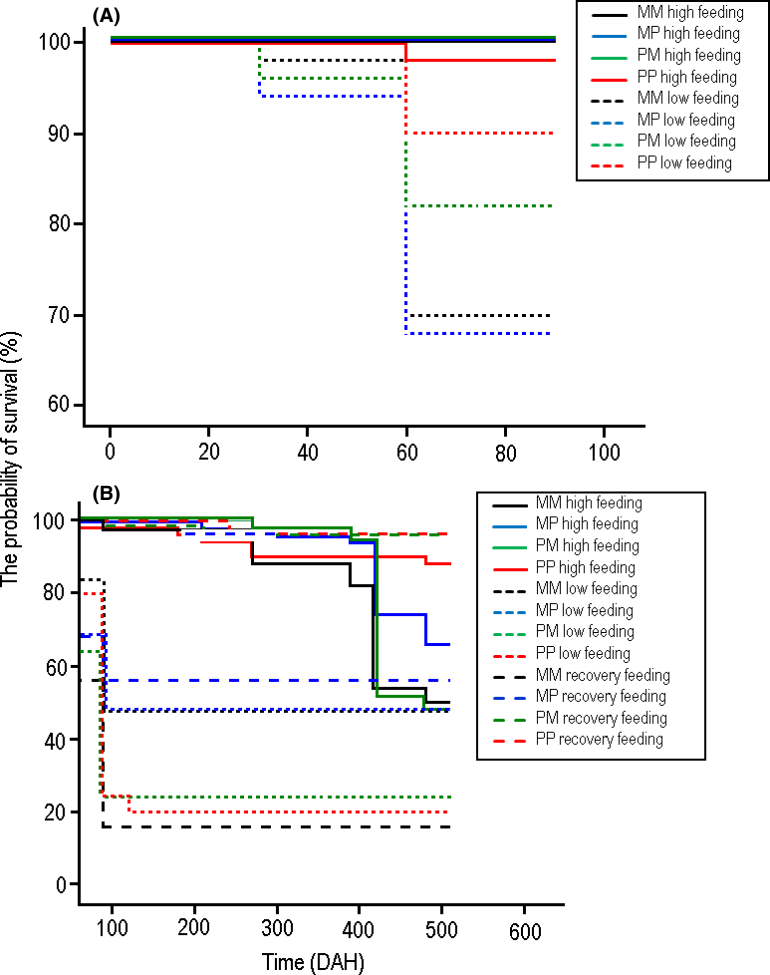
The probability of survival among four cross-types of nine-spined sticklebacks (A) before and (B) after initiation of the recovery feeding treatment. M = marine, P = pond. For the hybrids, the first abbreviation denotes origin of father, the second origin of mother.

Before the recovery feeding treatment was initiated on 90 DAH, the probability of survival differed significantly between high and low feeding treatments (*χ*^2^_1_ = 55.31, *P* < 0.0001). It was high (>98% Fig.3A) for all cross-types in the high feeding treatment and none of the cross-types differed significantly from each other (all *χ*^2^_1_ = 1.01, *P* ≥ 0.31). However, the probability of survival was lower in the low feeding treatment (90% PP > 84% PM > 70% MM > 68% MP; Fig.3A), and the pond fish had a significantly higher probability of survival than the marine and “hybrid” MP fish (all *χ*^2^_1_ > 5.06, *P* < 0.05). Growth rate (*SGR*) had a negative (*b* = −0.51) influence in the probability of survival (*χ*^2^_1_ = 171, *P* < 0.001). None of the interactions were significant (*χ*^2^ ≤ 0.45 × 10^−5^, *P* ≈ 1.00 in all cases).

After the recovery feeding treatment was initiated (91 DAH), feeding treatment, cross-type, and feeding treatment × cross-type interaction all had a significant influence on the probability of survival, whereas sex and other interaction effects were nonsignificant (Table3). In general, fish from the high feeding treatment tended to have higher survival probability than those from the low feeding treatment, and the crosses having a pond father tended to survive better than those having a marine father (Fig.3B). However, as indicated by the significant treatment × cross-type interaction, these generalizations hide significant heterogeneity.

**Table 3:** Cox regression analysis of the probability of survival among the four cross-types of *Pungitius pungitius* following recovery feeding treatment (91–510 d)

Source	df	*χ*^2^	*P*
Feeding treatment	2	46.08	< 0.0001
Cross-type	3	16.48	0.0016
Sex	1	9.06	0.1703
Growth rate	1	263.22	< 0.0001
Feeding treatment × cross-type	6	14.76	0.0222

Pairwise comparisons of survival probability within cross-types revealed that the marine fish subject to the recovery treatment had significantly lowered survival probability as compared to fish from both high and low feeding treatments (Appendix 6; Fig. 3B). In contrast, the pond fish from the low feeding treatment suffered from significantly lowered survival probability as compared to those from high or recovery feeding treatments, the latter of which experienced similar survival probabilities (Appendix 6; Fig. 3B). While the hybrids with marine fathers (MP cross) had similar intermediate (Fig.3B) survival probability in all treatments (Appendix 6), the hybrids with pond fathers had a significantly higher survival probability in recovery as compared to low and high feeding treatments, the latter of which did not differ in survival probability (Appendix 6; Fig. 3B). Pairwise comparisons of survival probability within treatments refined the picture (Appendix 7): in the recovery feeding treatment, all but one of the pairwise comparisons between cross-types were significant, whereas fewer significant differences were observed in the two other feeding treatments (Appendix 7).

### Probability of maturation

**Figure 4:**
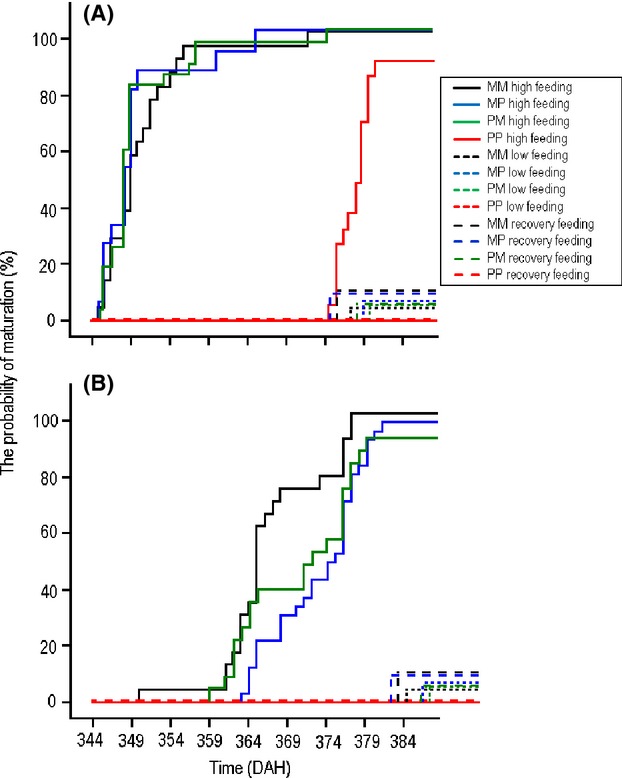
The probability of maturation among (A) male and (B) female nine-spined sticklebacks from four cross-types. M = marine, P = pond. For the hybrids, the first abbreviation denotes origin of father, the second origin of mother.

The probability of maturation was significantly influenced by the feeding treatment, cross-type, and sex (Table4). In general, fish from the high feeding treatment were more likely to mature than those from the recovery and low feeding treatments (84% high feeding > 23% recovery > 16% low feeding fish; Fig.4), and males (Fig.4A) were more likely to mature than females (Fig.4B) irrespective of feeding treatment and cross-type (Fig.4). However, a significant treatment × cross-type interaction revealed that feeding treatment effects were cross-type dependent (Table4). In the high feeding treatment, all of the marine fish and most of the hybrid fish (98% MP; 96% PM) but only 39% of the pond fish had matured by the end of the experiment (Fig.4). In the recovery feeding treatment, only 11% of the marine fish, 5–7% of the hybrid fish and none of the pond fish matured (Fig.4). Likewise, in the low feeding treatment, 5% of the marine fish, 5–6% hybrid fish and none of the pond fish matured by the end of the experiment (Fig.4). Hence, the general picture is that recovery feeding treatment did not restore the maturation probability of the initially starved fish anywhere close to the level observed among the fish in the high feeding treatment. All these effects are independent of growth rate (91–120 DAH) which had a significant positive effect on probability of maturation (significant main effect of growth rate, Table4), but apparently only in the high feeding treatment (significant feeding treatment × growth rate interaction, Table4).

**Table 4:** Cox regression analysis of the probability of maturation among the four cross-types of *Pungitius pungitius* after recovery feeding treatment (91–510 DAH)

Source	df	*χ*^2^	*P*
Feeding treatment	2	34.63	< 0.0001
Cross-type	3	47.89	< 0.0001
Sex	1	10.58	0.0085
Growth rate	1	120.93	0.0042
Feeding treatment × cross-type	6	13.05	0.0422
Feeding treatment × growth rate	2	9.31	0.0095
Cross-type × sex	3	21.19	< 0.0001
Cross-type × growth rate	3	2.79	0.4245
Sex × growth rate	1	3.77	0.0521
Cross-type × sex × growth rate	3	19.47	< 0.0001

## Discussion

In spite of the abundant scientific interest directed toward the study of compensatory growth, little is as yet known about its adaptive significance, associated costs, and population differentiation in it. The most salient findings of our study include evidence for compensatory growth responses in the pond population where individuals are destined to reach a large size, whereas in the marine population, where individuals are destined to reach a small size, compensatory growth responses were lacking. The observations that in both populations and their reciprocal crosses, the individuals subject to the recovery food treatment displayed full or even an overcompensation is also noteworthy. In other words, in spite of the early life food restriction, individuals from the recovery feeding treatment attained an equal or even larger size at the end of the experiment than those maintained in the high feeding treatment. However, the evidence was found also to indicate that the individuals subject to the recovery feeding treatment paid marked costs in terms of reduced probability of maturation and survival by the end of the experiment. In the following, we discuss these findings and their interpretations in relation earlier research, as well as how the observed population differences in responses to feeding treatments might relate to ecological differences between pond and marine nine-spined stickleback populations.

### Population differences in compensatory growth

Given that compensatory growth responses represent a form of adaptive plasticity likely to be favored by natural selection under certain, but not all, environmental conditions (e.g., Arendt 1997; Mangel and Munch 2005), geographic differences in environmental conditions selecting for differences in compensatory growth responses among populations would be expected. Yet, earlier studies comparing compensatory growth responses among different populations are rare (Purchase and Brown 2001; Schultz et al. 2002; Álvarez and Metcalfe 2007; Fraser et al. 2007). The results of our study add some fresh insights into this small literature. We found that the fish from the pond populations accelerated their growth above the routine levels when exposed to recovery treatment, while the fish from the fast growing marine population did not. This result does not accord with the observations that populations exhibiting highest routine growth rates are usually the ones that also show the evidence for compensatory growth responses (Schultz et al. 2002; Fraser et al. 2007). However, this contradiction may be more apparent than real. In the case of the nine-spined stickleback, pond fish have been selected to reach larger size than those from marine populations (e.g., Herczeg et al. 2012; Karhunen et al. 2014; Merilä 2013). There is also an evidence to suggest that fecundity selection favoring large females drives the evolution of large size in ponds (Herczeg et al. 2010). Conversely, high predation pressure from piscine predators in the marine environment is likely to select for early maturation at small size (Herczeg et al. 2012; Aikio et al. 2013), as well as select against strong compensatory growth responses because fast growth requires increased activity and movements which in turn increase risk of mortality through predation (Biro and Post 2008; Biro and Stamps 2008). Other possible factors favoring the evolution of compensatory growth responses in ponds relate to the high seasonality and fluctuating feeding conditions in the oligotrophic northern pond ecosystems: short growth seasons and periods of food shortage relating to large fluctuations population density and food availability (Merilä 2013) may favor mechanisms buffering the growth against perturbations. Likewise, low extrinsic mortality allowing pond fish to attain old ages (Herczeg et al. 2009; DeFaveri et al. 2014) could select for investments made toward repair and maintenance of soma. While such investments are usually viewed to trade-off with resources available for growth (Cichon 1997; Metcalfe and Monaghan 2003), it seems not implausible that compensatory growth responses could also be viewed as a form of investment on soma. In particular, the lack of compensatory growth responses in fast growing and short-lived marine nine-spined sticklebacks, but their presence in slow growing and long-lived pond counterparts contradicts the idea of trade-off between investment in growth and maintenance unless one views compensatory growth as a form of self-maintenance.

Significant population differences in compensatory growth responses were observed in Atlantic silversides (*Menidia menidia*, Schultz et al. 2002) and in the Atlantic salmon (*Salmo salar*, Fraser et al. 2007), but not in the cod (*Gadus morhua*, Purchase and Brown 2001). In the case of the silversides, the stronger compensatory growth responses in the high as compared to low latitude populations were hypothesized to result from selection stemming from short breeding in the north, favoring mechanisms allowing individuals to exploit “windows of opportunity” to gain size. Likewise, Fraser et al. (2007) found that individuals from the long-distance migrating population salmon exhibited stronger compensatory response to food deprivation than those from the short-distance migrating population. This was suggested to reflect the needs for long-distance migrants to reach a large body to offset the energetic costs of long migration and to compensate for the shorter time they spend on feeding grounds. Although these inferences accord with the findings of our study, neither of these previous studies controlled for possible initial size differences among the control and treatment fish, making it difficult to judge whether the observed growth responses actually represented compensatory growth (*cf*. Nicieza and Álvarez 2009; Jobling 2010, see below).

### Genetic basis of recovery growth potential

Results of an earlier study (Ab Ghani et al. 2012) using the fish from the high feeding treatment revealed that the body size differences between pond and marine populations appear to have mainly an additive genetic basis. Our results support this conclusion but show that this inference may be sensitive to environmental conditions under which the fish were reared. In all feeding treatments, fish from pure pond crosses were the largest, and those from pure marine crosses the smallest. However, whereas the reciprocal hybrids were intermediate in size to the two pure crosses in the high and recovery feeding treatments (Figs.1, 2), the growth-deprived fish from the low feeding treatment showed clear signs of genetic dominance: the size in both reciprocal hybrid crosses converged toward that of the pure pond fish. Further, the strong cross-type by feeding interactions in growth responses were indicative of genetic differences in how fish from different populations respond to food deprivation. For instance, the marine fish from the recovery treatment showed over compensation,while the pond fish in this treatment showed full compensation. However, although growth trajectories and body sizes at the end of the experiments in high and recovery feeding treatments conformed to what would be expected under an additive mode of inheritance (i.e., hybrids intermediate to pure crosses), the initial (90–201 DAH; Fig.2A), growth responses to removal of food restriction in the recovery treatment showed clear signs of genetic dominance. Namely, while pure pond and both hybrid crosses followed roughly a similar growth trajectory, the pure marine fish showed no evidence for a compensatory growth response. Nevertheless, the fact that final sizes of the fish from the recovery treatment rebounded to the pattern seen among the fish in the high feeding treatment indicates strong resilience in growth patterns toward environmental perturbations.

The observed cross-type-specific patterns of growth resemble the inverse of that seen in age at maturation in these populations: pure marine and both hybrid crosses have high and similar probability of maturing early, whereas the opposite is the case for pure pond fish (Ab Ghani et al. 2013a). However, the detailed analysis of growth responses following the cessation of food restriction revealed evidence for maternal effects mediating the recovery growth responses. Namely, both growth rates and size-adjusted growth rates were considerably higher for pure pond crosses and hybrid crosses with pond mothers than for pure marine crosses and hybrid crosses with marine mothers. This strongly suggested a female component to growth responses, possibly mediated through some pre- or postnatal maternal contributions to offspring development. While this may not be surprising given that maternal effects on offspring phenotypes and growth are ubiquitous (Green 2008), it is interesting that such effects were manifested in conjunction with the feeding treatment responses. In fact, these influences were still perceivable at the end of the experiments in the tendency of the mean body size of the hybrid crosses from the recovery treatment to resemble that of their respective maternal pure cross (Fig.1). Hence, these results suggest that both genetic and maternal effects influence recovery growth responses.

### The costs of recovery growth

The observations that growth rates are rarely maximized in the wild and that organisms grow at rates below their physiological potential has lead to the realization that there must be costs involved with fast growth rates (e.g., Mangel and Munch 2005; Dmitriew 2011). Here, we focussed on the potential intrinsic costs of compensatory growth by comparing maturation and survival probability of individuals from different treatments. In respect to maturation probability, we found evidence for the elevated cost of growth compensation: fish subject to the recovery treatment had a lower probability of maturation than fish from the high feeding treatment. However, this interpretation could be challenged by the observation that the probability of maturation in the recovery treatment was similar to that in the low feeding treatment. In other words, the food restriction itself could be the cause for the delayed maturation as shown for instance in guppies (Auer 2010; see also: Lee et al. 2012). The probability of maturation in fish has also been shown to be influenced by temperature, independent of growth (Chimlevskii 1996; Kuparinen et al. 2011). For instance, Chimlevskii (1996) observed that the transfer from lower to higher temperature resulted in growth compensation, but maturation was still delayed as compared to fish grown in higher temperature. Likewise, in the Atlantic salmon, food deprivation followed by unrestricted feeding has been shown to lead to decreased probability of maturation as compared to controls maintained in unrestricted feeding (Reimers et al. 1993; Morgan and Metcalfe 2001). Hence, these results in combination with results from our study suggest that unfavorable environmental conditions delay maturation, and that opportunity to compensatory growth may fail to erase this effect. Whether compensatory growth itself can delay maturation remains contentious.

Viewing delayed maturation as a cost could be challenged also from the grounds that postponed maturation usually translates to increased size and thereby also increased fecundity at maturity (Roff 1992; Stearns 1992). We did not asses fecundity in this study, but as body size is positively correlated with clutch and egg size in this species (Herczeg et al. 2010), the fact that individuals from the recovery treatment were equally large – or even larger in the case of the crosses showing overcompensation – than individuals from high feeding treatment suggests that they also had increased fecundity at maturity. However, this reasoning assumes that food restriction and subsequent recovery growth do not carry any unhidden costs. Yet, compensatory growth can have negative impacts on reproductive traits and physiology if it decreases the energy available for their maintenance and development, or if it interferes with the allocation of energy or nutrients to reproductive traits. In fact, there is some evidence suggesting that recovery growth can have negative impacts on nonreproductive (Ricklefs et al. 1994; Arendt et al. 2001; Robinson and Wardrop 2002; Arendt 2003), as well as on reproductive traits (Auer et al. 2010; Lee et al. 2012; Ab Ghani and Merilä 2014). Recovery growth could also negatively affect reproduction if it increases metabolic needs for growth and thereby decreases the amount of energy available for reproduction.

Although many studies have sought to quantitate costs of compensatory growth (reviewed in Ali et al. 2003), few have looked for or found any evidence for costs in terms of survival probability (but see: Billebeck et al. 2001; Carlson et al. 2004; Johnsson and Bohlin 2006; Inness and Metcalfe 2008; Lee et al. 2013). We found that the compensatory treatment induced increased mortality relative to high feeding treatment, but this effect was cross-type dependent. Both crosses with marine fathers experienced increased mortality in the recovery as compared to the high feeding treatment, whereas crosses with pond fathers showed no difference or even reduced mortality in the recovery treatment. This pattern does not support the possibility that maternal effect influences would have been important determinants of the mortality patterns, but rather, is indicative of nonadditive genetic effects. However, regardless of the underlying cause for this heterogeneity, it seems clear that in the case of the pure crosses, the effect of compensatory feeding was to lower the survival probability for the marine fish (mortality rate: recovery > low ≈ high) and restore it in the pond fish (mortality rate: low > high ≈ recovery).

### Measurement of compensatory growth

The literature focussed on compensatory growth responses is voluminous (reviewed in: Ali et al. 2003). However, the actual evidence supporting compensatory growth as an important and widespread adaptive mechanism in mitigating negative fitness consequences of early life growth deprivation might not be as widespread as the literature lends to believe. Namely, there is a considerable conceptual and methodological confusion as to what actually constitutes evidence for compensatory (as opposed to normal, catch-up, and recovery) growth, and how the compensatory responses should be compared to controls without “accumulating false empirical support” (*cf*. Nicieza and Álvarez 2009). In addition, many if not most studies of compensatory growth responses have focussed on body mass increments which may confound changes in body composition and energy reserves with growth. In this study, these problems were avoided using a linear measure of size, and by comparing growth responses among treatments using methods which account for size dependency in growth responses (Nicieza and Álvarez 2009; Jobling 2010). The results comparing size corrected and uncorrected measures of growth rates reinforce the view that failure to correct for initial size differences can lead to false conclusions about the occurrence of compensatory growth: much of the differences in compensatory growth responses were erased once initial size was corrected for. Nevertheless, the results and conclusions appeared to be robust in respect to growth responses in the pond population. The conclusion was reinforced also by the fact that in several instances, the growth trajectories of deprived-recovery fish actually overshoot those of the controls at the end of the experiments. Examples of over compensatory responses are very rare in the literature (Ali et al. 2003), and the sheer fact that they were observed in this study is itself a strong signal that the observed responses were not confined to just subtle differences in instantaneous growth rates, but actually lead to qualitative differences in body size at the end of the experiments. Finally, we note that the rather drastic mortality at time of application of the recovery feeding treatment among the low and recovery feeding treatment fish (Fig.3B) is explainable by the fact that the fish in both these treatments experienced challenging low feeding treatment until 90 DAH, after which half the low feeding treatment fish were assigned to recovery treatment (see methods). However, as all cross-types (*viz*. MM, MP, PM & PP) were treated in the same fashion, the possible implications of this mortality for the interpretations of the results should be minimal. This at least for comparisons across the cross-types and any comparisons restricted to the high feeding treatment.

## Conclusions

The results demonstrate the occurrence of compensatory growth in response to early life food restriction in a pond population of nine-spined sticklebacks, as well evidence for significant catch-up (but not for compensatory) growth in a marine population. In other words, although marine fish were not observed to accelerate their growth in response to removal of food restriction above routine levels, as was the case for the pond fish, fish from both populations compensated for early growth restriction by reaching similar (pond) or larger (marine) sizes than their conspecifics grown in unlimited food rations. Experiments with pure and hybrid crosses further indicated that the observed population differences in growth responses had at least a partially genetic basis. We also observed clear evidence for costs for early life food restriction and that these costs differed among populations. Similar studies conducted in multiple population contexts are as yet rare and conducted without controlling for size dependency in growth responses. Likewise, given that only one earlier study (Hayward et al. 1997) has found evidence for overcompensation in response to food restriction, our finding of size overcompensation in the marine sticklebacks is noteworthy. Further studies utilizing controlled breeding designs able to disentangle genetic and maternal effects on compensatory growth responses in an intrapopulation context, as well as experiments utilizing replicate pond and marine populations can provide possible further avenues to understand evolutionary potential and significance of compensatory growth responses, and their costs.

## Figures and Tables

**Figure 5:**
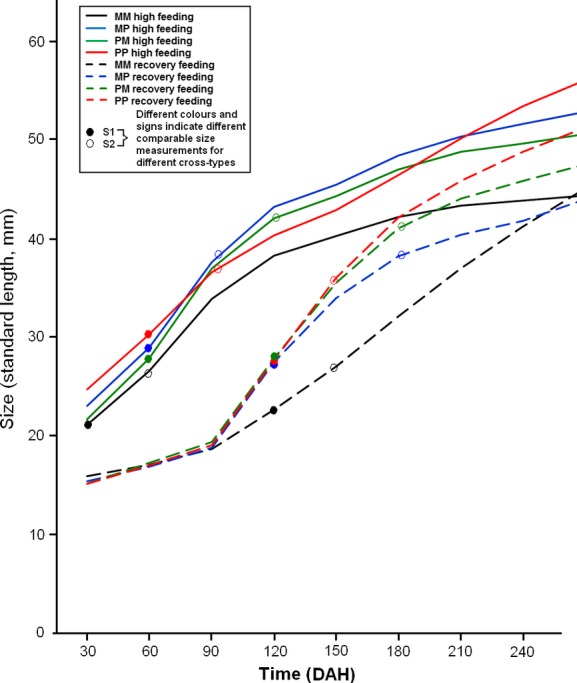
Definition of the comparable size points (S1–S2) in high and recovery feeding treatments which were used to compare specific growth rates (*SGR*) in the “asynchronous” approach. Note that exact matching of comparable points was not always possible because of fixed time intervals used for measurements.

## Appendix 1: Comparison of growth rates among treatment groups using “asynchronic approach”

Apart from the “synchronic” approaches reported in the main text, we also utilized an “asynchronic” approach of Nicieza and Álvarez (2009) to compare the growth rates between high and recovery feeding treatments. The rationale in this approach is to compare growth rates between feeding treatments over “comparable time points”, that is when the individuals in the control (in this case, high feeding) treatment are at a comparable size to the treatment fish at the beginning of the recovery growth phase. The comparable time points and sizes were determined and defined graphically (see Fig. 5A1 for details). However, the problem with our data was that individuals at the beginning of the recovery growth phase were often smaller than individuals from the high feeding treatment for the time period we had growth data (Fig. 5A1). Hence, to use this method, we were forced to compare growth rates that did not necessarily encompass the fastest phase of recovery growth. Yet the results were concurrent with those obtained with other approaches.

This approach controls for initial size differences among treatment groups utilizing comparable size points as depicted in FigureA1 below. The asynchronous approach was applied on specific growth rates (*SGR*s) at comparable sizes using a GLMM. Here, the comparable measurements form high and recovery feeding treatments were used to estimate *SGR* which was used as a dependent variable, while feeding treatment, cross-type, and sex were treated as fixed factors. Family nested within cross-type was added as a random factor. All two- and three-way interactions between fixed factors and the single explanatory variable were included in the initial model. A backward stepwise model selection based on the *P *<* *0.05 criterion (see main text).

Comparison of *SGR* between high and recovery feeding treatments with a GLMM confirmed that at comparable sizes, fish from the recovery treatment had significantly higher growth rates than those from high feeding treatment (Feeding treatment: *F*_1,246_ = 2.75, *P* < 0.05; TableA1). A significant feeding treatment × cross-type interaction (TableA1) occurred due to the lack of compensatory growth response in MM cross (Fig. A1; TableA1).

**Table A1:** GLMM of specific growth rates (SGRs) as estimated from asynchronous approach at comparable time points (S1 and S2) for all cross-type in high and compensatory feeding treatments. The comparable time points are defined in Fig. A1. Family within cross-type was added as random factor. Significant effects in boldface. df_1_ = numerator degrees of freedom, df_2_ = denominator degrees of freedom

Source	df_1_, df_2_	*F*	*P*
**Feeding treatment**	**1246**	**2.75**	**0.0498**
**Cross-type**	**152**	**3.17**	**0.0318**
Sex	1235	0.44	0.5098
**Feeding treatment ×cross-type**	**3239**	**29.11**	**< 0.0001**
**Feeding treatment × sex**	**1233**	**5.08**	**0.0251**
Cross-type × sex	3237	2.13	0.0968
Feeding treatment × cross-type × sex	3228	1.74	0.1601

## Appendix 2: Details about sample sizes in survival and maturation analyses

Survival: Before recovery feeding started, a total of 352 individuals were alive, comprised of 199 individuals from the high feeding (MM: 50, MP: 50, PM: 50, and PP: 49) and 153 individuals from the low feeding (MM: 42, MP: 37, PM: 43 and PP: 31) treatment. Of the 48 dead individuals, one was from the high feeding (PP: 1) and 47 were from the low feeding treatment (MM: 15, MP: 16, PM: 10, and PP: 6). After recovery feeding was initiated, a total of 227 individuals survived until the end of the experiments, comprised of 126 individuals from the high feeding (MM: 25, MP: 33, PM: 24, and PP: 44), 69 individuals from the recovery feeding (MM: 22, MP: 17, PM: 16, and PP: 14) and 32 individuals from the low feeding (MM: 13, MP: 10, PM: 6, and PP: 3) treatment. Of the 125 individuals that died during the second time interval, 73 were from the high feeding (MM: 25, MP: 17, PM: 26, and PP: 5), 31 were from the recovery feeding (MM: 3, MP: 8, PM: 9, and PP: 11) and 21 were from the low feeding (MM: 4, MP: 2, PM: 12, and PP: 3) treatment.

Maturation: Timing of maturation was recorded starting from the day when the first artificial hibernation ended (344 DAH) and continued until 510 DAH. During this time interval, records were available for a total of 288 surviving individuals (high feeding: 187 individuals [MM: 44, MP: 48, PM: 49, and PP: 46], low feeding: 35 individuals [MM: 12, MP: 12, PM: 6, and PP: 5], and recovery feeding: 66 individuals [MM: 4, MP: 14, PM: 24, and PP: 24]) which included both mature and immature individuals.

## Appendix 3

Pairwise comparison of the mean body size among four-nine-spined stickleback cross-types in different feeding treatments at the end of the experiment (510 DAH) as revealed by post hoc tests. M = marine; P = Pond. For the hybrids, the first abbreviation denotes origin of father, the second denotes origin of mother. Significant effects in boldface. For all effects, df = 379

**Table:**

	Marine (MM)	“Hybrid” MP	“Hybrid” PM	Pond (PP)
High: low	**2.14****	**2.89****	**2.92****	**9.83*****
High: recovery	**2.83****	1.77	**5.79*****	0.93
Low: recovery	**2.47****	**2.98****	**7.55*****	**9.33*****

* *P *<* *0.05

** *P *<* *0.01

*** *P *<* *0.001.

## Appendix 4

Results of GLMM of growth increment (*k*) between 91 and 120 DAH among four cross-types of *Pungitius pungitius* in high and recovery feeding treatments. A backward stepwise model selection was applied, and nonsignificant terms are shown as seen at removal. Significant effects in boldface. SL90 = standard length at 90 DAH. df_1_ = numerator degrees of freedom, df_2_ = denominator degrees of freedom

**Table:**

Source	df_1_, df_2_	*F*	*P*
**Feeding treatment**	**1235**	**4.38**	**0.0135**
**Cross-type**	**3212**	**6.56**	**< 0.0001**
Sex	1237	3.24	0.0732
SL90	1241	0.63	0.4277
**Feeding treatment ×cross-type**	**3212**	**3.81**	**0.0109**
**Feeding treatment × sex**	**1234**	**3.98**	**0.0471**
Feeding treatment × SL90	1231	0.59	0.4449
**Cross-type × sex**	**3236**	**4.70**	**0.0033**
**Cross-type × SL90**	**3210**	**5.85**	**0.0007**
Sex × SL90	1237	3.68	0.0564
**Feeding treatment × cross-type × sex**	**3232**	**4.25**	**0.0060**
Feeding treatment × cross-type × SL90	3225	1.52	0.2091
Feeding treatment × sex × SL90	1231	1.10	0.2964
**Cross-type × sex × SL90**	**3235**	**4.62**	**0.0037**

## Appendix 5

Repeated measures general linear mixed model with size at 90 and 102 DAH as response variables, and feeding treatment, cross-type, and sex as fixed effects. A backward stepwise model selection was applied, and nonsignificant terms are shown as seen at removal significant effects in bold. df_1_ = numerator degrees of freedom, df_2_ = denominator degrees of freedom

**Table:**

Source	df_1_, df_2_	*F*	*P*
**Feeding treatment**	**1504**	**1322.95**	**< 0.0001**
**Cross-type**	**3498**	**21.79**	**< 0.0001**
**Sex**	**1488**	**16.29**	**< 0.0001**
**Repeat (time)**	**1471**	**342.86**	**< 0.0001**
**Feeding treatment × cross-type**	**3494**	**11.29**	**< 0.0001**
Feeding treatment × sex	1487	1.60	0.2070
**Feeding treatment × repeat**	**1471**	**95.34**	**< 0.0001**
Cross-type × sex	3488	2.79	0.0402
Cross-type × repeat	3471	0.77	0.5107
Sex × repeat	1471	0.06	0.7991
**Feeding treatment × cross-type × sex**	**3487**	**4.94**	**0.0022**
Feeding treatment × cross-type × repeat	3468	1.23	0.2988
Feeding treatment × sex × repeat	1467	0.21	0.6454
Cross-type × sex × repeat	3464	0.35	0.2753

## Appendix 6

Pairwise comparison of the probability of survival during 91–510 DAH in different feeding treatments among four *Pungitius pungitius* cross-types based on Cox regression analyses. M = marine P = Pond. For the hybrids, the first abbreviation denotes origin of father, the second denotes origin of mother. Significant effects in boldface. For all effects, df = 1

**Table:**

	Marine (MM)	Hybrid MP	Hybrid PM	Pond (PP)
High: low	0.03	1.56	3.06	**41.67*****
High: recovery	**6.75****	0.07	**16.67*****	0.49
Low: recovery	**4.50***	0.32	**24.08*****	**35.34*****

* *P *<* *0.05

** *P *<* *0.01

*** *P *<* *0.001.

## Appendix 7

Pairwise comparison of the probability of survival after recovery feeding treatment (91–510 DAH) among cross-types of *Pungitius pungitius* in all feeding treatments based on Cox regression survival analyses. M = marine; P = pond. For the hybrids, the first abbreviation denotes origin of father, the second origin of mother. Significant effects in boldface. For all effects, df = 1

**Table:**

	High feeding treatment	Recovery feeding treatment	Low feeding treatment
MM: PP	**15.15*****	**29.30*****	**8.04****
MM: MP	2.01	**8.49****	0
MM: PM	0.04	**29.30*****	2.17
MP: PM	2.61	**7.46****	2.17
PP: MP	**5.65***	**7.46****	**8.04****
PP: PM	**16.59*****	0	1.34

* *P *<* *0.05

** *P *<* *0.01

*** *P *<* *0.001.
